# Transcriptional profiles of PBMCs from pigs infected with three genetically diverse porcine reproductive and respiratory syndrome virus strains

**DOI:** 10.1007/s11033-018-4204-x

**Published:** 2018-06-07

**Authors:** Marzena Rola-Łuszczak, Magdalena Materniak-Kornas, Aneta Pluta, Katarzyna Podgórska, Jens Nielsen, Tomasz Stadejek, Jacek Kuźmak

**Affiliations:** 1 Department of Biochemistry, National Veterinary Research Institute, Partyzantow 57, 24-100 Pulawy, Poland; 2 Department of Swine Diseases, National Veterinary Research Institute, Partyzantow 57, 24-100 Pulawy, Poland; 3 National Veterinary Institute, Technical University of Denmark, Kalvehave, 4771 Lindholm, Denmark; 4 Department of Pathology and Veterinary Diagnostic, Faculty of Veterinary Medicine, Warsaw University of Life Sciences, Nowoursynowska 159C, 02-776 Warsaw, Poland; 5 Department of Microbiological Diagnostics and Virology, Statens Serum Institut, 5 Artillerivej, 2300 Copenhagen, Denmark

**Keywords:** PRRSV, Pig, Transcriptome analysis, Host immune response

## Abstract

Porcine reproductive and respiratory syndrome virus is the cause of reproductive failure in sows and respiratory disease in young pigs, which has been considered as one of the most costly diseases to the worldwide pig industry for almost 30 years. This study used microarray-based transcriptomic analysis of PBMCs from experimentally infected pigs to explore the patterns of immune dysregulation after infection with two East European PRRSV strains from subtype 2 (BOR and ILI) in comparison to a Danish subtype 1 strain (DAN). Transcriptional profiles were determined at day 7 post infection in three tested groups of pigs and analysed in comparison with the expression profile of control group. Microarray analysis revealed differential regulation (> 1.5-fold change) of 4253 and 7335 genes in groups infected with BOR and ILI strains, respectively, and of 12518 genes in pigs infected with Danish strain. Subtype 2 PRRSV strains showed greater induction of many genes, especially those involved in innate immunity, such as interferon stimulated antiviral genes and inflammatory markers. Functional analysis of the microarray data revealed a significant up-regulation of genes involved in processes such as acute phase response, granulocyte and agranulocyte adhesion and diapedesis, as well as down-regulation of genes enrolled in pathways engaged in protein synthesis, cell division, as well as B and T cell signaling. This study provided an insight into the host response to three different PRRSV strains at a molecular level and demonstrated variability between strains of different pathogenicity level.

**Electronic supplementary material:**

The online version of this article (10.1007/s11033-018-4204-x) contains supplementary material, which is available to authorized users.

## Introduction

Porcine reproductive and respiratory syndrome (PRRS) is a viral disease of significant economic impact on a swine industry worldwide. Clinically the disease manifests by reproductive disorders in sows and respiratory lesions and poor growth performance in growing pigs. The etiological agent of the disease, PRRS virus (PRRSV) is an enveloped, positive-sense single-strand RNA virus classified in the order *Nidovirales*, family *Arteriviridae*. Two genotypes, Type 1 and Type 2, sharing approximately 60% of genetic similarity were described [1]. A newly proposed classification denotes both types as different species within *Arteriviridae* family (https://talk.ictvonline.org/taxonomy/). Type 1 can be further divided into at least four genetic subtypes, namely Pan-European subtype 1 and subtypes 2, 3 and 4 represented by strains circulating in Eastern European countries [2]. Gathering evidences suggest the existence of additional subtypes. However, the genetic subtyping based on a small genomic fragment of ORF5 and ORF7 can be affected by genetic recombination [3–5].

PRRSV shows a remarkably high degree of genetic variation translating into high antigenic and pathogenic variability. High frequency of recombinations also contributes to occasional changes of biological properties and virulence level [6, 7]. The most striking example is a highly pathogenic Type 2 PRRSV variant with 30 aa discontinuous deletion within nsp2 protein that emerged in 2006 in China devastating swine industry in several Asian countries [8, 9]. Recent animal infection studies indicated that some East European strains of PRRSV Type 1 have been characterized by higher pathogenicity compared to the mild syndrome produced by infection with subtype 1 [10–12].

The differences in pathogenicity between PRRSV strains were hypothesized to originate from different degrees of immunomodulatory properties. The virus utilizes a range of mechanisms to influence the immune response including weak stimulation of interferon production and slow development of cell-mediated immunity, inhibition of the expression of pro-inflammatory cytokines and weak and delayed neutralizing response [13]. As a result, the course of infection may advance to a chronic stage lasting even up to 21 weeks [14]. Insufficient stimulation of an immune response and high level of genetic variability also constitute key problems in the development of efficient vaccines. None of the currently used vaccines provides full protection against infection and their effectiveness is often compromised towards heterologous strains [15]. Deeper knowledge on the core mechanisms of host-pathogen interactions and the relevance of genetic diversity is necessary to facilitate the development of efficient tools for PRRS control.

Interestingly, previous studies revealed that the pattern of cytokines expression after infection with prototype subtype 3 strain Lena differed compared to subtype 1 strains [16, 17]. Together with observations from a study on another subtype 3 strain SU1-bel [11], where greater interferon-γ response was noted, those results suggested that observed higher pathogenicity may be a result of enhanced inflammatory immune response.

Therefore the aim of the present study was to explore and compare the patterns of immune response after infection with two East European PRRSV strains, genetically classified as subtype 2, but showing different pathogenicity, and a classical Danish subtype 1 strain. For this, microarray-based transcriptomic analysis of PBMCs collected at the peak of viremia from pigs infected with both subtypes was performed.

## Materials and methods

### Animals and infection with PRRSV strains

The blood samples used in the study were collected during an animal challenge experiment described elsewhere [18]. The pigs used in the study originated from a high health pig herd maintained by the Institute and were free from infections with the following pathogens: encephalomyocarditis virus, hepatitis E virus, porcine circovirus type 1 and type 2 viruses, porcine cytomegalovirus, porcine epidemic diarrhoea virus, porcine parvovirus type 1, porcine respiratory coronavirus, PRRSV Type 1 and Type 2, influenza A virus, transmissible gastroenteritis virus, *Actinobacillus pleuropneumoniae, Bordetella bronchiseptica, Brachyspira hyodysenteriae, Brachyspira pilosicoli* and *Brucella* spp, using in-house standard diagnostic methods. In short, piglets from three sows were divided into four experimental groups, ensuring that each litter was equally represented in every group. Three groups of 8-week-old pigs were infected with a PRRSV Type 1 strains including subtype 1 strain 18,794 (DAN) isolated in 1993 in Denmark [19], a Russian isolate ILI6 (ILI) and a Belarusian isolate BOR59 (BOR) from 2009, each classified as subtype 2 strain based on ORF5 sequence, respectively. Pigs were inoculated intranasally with 2 ml of virus inoculum prepared based on 5th (titre of 5.4 log10 TCID50/ml), 3rd (titre of 6.8 log10 TCID50/ml) and 2nd (titre of 4.4 log10 TCID50/ml) passage in porcine alveolar macrophages culture for DAN, ILI and BOR strains respectively. The fourth, PRRSV- negative control group, was sham-inoculated with 2 ml of Eagle’s medium. Individual pigs were subjected to daily clinical examination and measurement of rectal body temperature. The severity of clinical lesions was assessed based on a scoring system adapted to PRRS [18]. An overall well-being, respiration, eye disorders and appetite were scored as 0 (normal condition) to 3 (severe disorder). The scores for individual pigs were added up to a cumulative clinical score (CS) per day. During the experiment no mortality was recorded and none of the animals displayed the acute clinical signs defined as endpoint criteria. Euthanasia of the pigs was performed on 22–24 DPI by intravenous injection of pentobarbiturate (50 mg/kg) followed by exsanguination by cutting arteria axillaris.

Blood samples were collected on − 2, 0, 3, 7, 10, 14 and 21 days post infection (dpi) from the anterior vena cava. The peak of the level of viremia in all experimental groups was observed at 7th dpi. Moreover, at the same time point the most severe clinical lesions (measured by an objective clinical scoring system) were observed in BOR group, while in other groups no or very mild clinical lesions were observed described in details in Stadejek et al. [18]. Therefore, samples collected at 7th dpi were submitted for further microarray study. EDTA—stabilized blood samples were collected from five pigs from each group and peripheral blood mononuclear cells (PBMC) were isolated by gradient density centrifugation using Histopaque 1077 (Sigma-Aldrich), frozen and stored at − 80 °C until further processing.

### Microarray analysis

Transcriptional profiles of PBMC of infected and control piglets were analyzed using oligonucleotide microarrays specific for Sus Scrofa from Agilent, ID 062763, 8 × 60 K format. Total RNA was extracted from PBMCs using Rneasy kit (QIAGEN) and the preparations with RNA Integrity Number (RIN) from 7.5 to 10 were used. As the experiment was performed using pigs of highly uniform genetic and environmental background, RNA samples representing template of animals from each control and experimental group were pooled. Each pool was processed in four repeats using Two-Color Microarray-Based Gene Expression Analysis, Low Input Quick Amp Labeling-Agilent Technologies. Briefly, 50 ng of total RNA (equal amount of RNA of each animal were pooled) was reverse transcribed to generate cDNA and then transcribed into Cy3-labelled cRNA (samples obtained from control animals) and into Cy5-labelled cRNA (samples obtained from infected animals). After purification of labelled RNA (Qiagen RNeasy Kit), the yield (ng of cRNA) and specific activity (pmol of Cy3 or Cy5/µg of cRNA) were quantified using Nanophotometr Pearl (IMPLEN). Hybridization was performed by preparing a target solution containing 300 ng Cy5-labeled pool cRNA from infected animals, 300 ng Cy3-labeled pool cRNA of uninfected animal and then fragmentation buffer was added and incubated at 60 °C for 30 min. After stopping fragmentation, samples were hybridized on Agilent arrays for 18 h at 65 °C in Agilent hybridization chambers in an Agilent hybridization oven rotating at 10 rpm. After hybridization the arrays were subsequently washed with ‘GE wash buffer 1’ for 1 min at room temperature, ‘GE wash buffer 2’ for 1 min at approximately 37 °C, each chamber washed twice. After washing, slides were scanned using Agilent G2505C US10353831. Images obtained after scanning were analyzed using Agilent Feature Extraction software (version 10.7.3.1). A detailed analysis including filtering of outlier spots, background subtraction from features and dye normalization (linear and LOWESS) was performed. The raw and processed data discussed in the study have been deposited in NCBI’s Gene Expression Omnibus (GEO) with accession number GSE95213.

### Microarray gene functional analysis

Analysis of microarray results was carried out using the GeneSpring GX10 expression analysis software (Agilent Technologies). The genes were determined to be differentially expressed if the fold change (FC) was greater than 1.5 in up- or down-regulation. Statistical differences in gene expression were determined with a Student’s t-test at p ≤ 0.05 and also a false discovery rate (FDR) < 0.05 was used as a threshold. The lists of candidate genes identified by GeneSpring analysis were uploaded to the Ingenuity Pathway Analysis program (IPA; https://www.qiagenbioinformatics.com/products/ingenuity-pathway-analysis/) to identify most biologically relevant changes. Separate lists of up- and down-regulated genes were analyzed using Canonical Pathway analysis, which used right-tailed Fisher’s Exact Test to identify pathways enriched in the gene set compared to the reference set of genes which included all genes in the human genome.

### RT-qPCR

To validate the microarray results, six genes (OAS1, CXCL2, Il-8, CXCL10, FOS and IL-4) were selected to include genes that were found to be differentially expressed in each group of infected animals. Moreover, these genes were previously described in the context of PRRSV infection. Results were normalized using β-actin as a reference gene. These quantitative real-time reverse-transcription PCRs were carried out using the pooled RNA samples subjected to microarray study. Furthermore, to validate whether analysis of pooled samples reflects the gene expression in the individual specimens, also the relative expression of four selected genes (OAS1, CXCL2, IFN-α and IFNβ) was assessed in RNA from the individual pigs by RT-qPCR. The list of primers used in the study is shown in Table 1. Primers specific for the β-actin gene were used as described elsewhere [20], while the primers for target genes were designed using Primer 3 Plus bioinformatics tool (http://primer3plus.com/cgi-bin/dev/primer3plus.cgi). Similarly as for microarray experiment, RNA samples from each control and experimental group were pooled ensuring the same concentration of RNA from each animal. Then 1.5 µg of each pooled sample were digested with DNase I Amplification Grade (Invitrogen) and after reverse transcribed using NG dART RT kit (EURX). The synthesis of cDNA was performed at 47 °C for 50 min. The qPCR was performed in a Rotor Gene Q (Qiagen). Each reaction mix contained 80 ng of cDNA, 2x QuantiTect SYBRGreen Mix (Qiagen) and 10 µM of forward and reverse primers specific for each tested gene and adjusted to a total volume of 25 µl. Every qPCR reaction was performed in duplicate (technical replicates). The efficiency of each reaction was determined based on a serial dilution of cDNA template (100, 10 and 1 ng) and remained within a 90–110% range. Relative gene expression levels were calculated using E-method described by Pfaffl [21] and fold-change units were calculated by dividing the normalized expression values coming from infected animals by the normalized expression values in the controls. The same procedure was applied for individual samples where 80 ng of each individual RNA sample was used.

**Table 1:** List of primers used in RT-qPCR

Gene	Reference sequence ID	Primer sequence (5′–3′)
β-actin	XM_003357928.2	F: CTCGATCATGAAGTGCGACGT R: GTGATCTCCTTCTGCATCCTGTC [20]
OAS1 2′-5′-Oligoadenylate synthetase 1, 40/46 kDa	NM_214303.1	F: CTTTGCATCTTCTGGGAAGC R: AGGCCTGGGTTTCTTGAGTT
CXCL2 Chemokine (C-X-C motif) ligand 2	NM_001001861.2	F: CCCTTGGACATTTTATGTCTTCC R: GGACAGAGCGGAAACACAGT
Il-8 Interleukin 8	NM_213867.1	F: AGGAAAAGTGGGTGCAGAAG R: CCACGGAGAATGGGTTTTTG
CXCL10 Chemokine (C-X-C motif) ligand 10	NM_001008691.1	F: CCACTTTGGGACTTAATCGAAG R: AGAAGCCCACGGAGTAAAGA
FOS FBJ murine osteosarcoma viral oncogene homolog	AJ132510.1	F: TTTCCTTCGGCATCAATGT R: CATTCAGACCACCTCAC
IL-4 Interleukin 4	NM_214123.1	F: GACAGGAACCTGAGCAGCA R: TCGTCTTTAGCCTTTCCAAGA
IFNα Interferon-alpha	NM_001166319.1	F: CCTGTGCCTGGGAGATC R: CTCCTTCTTCCTGAATCTGTC
IFNβ Interferon-beta	KF414741.1	F: CAGTACCTGAAGTCCAAGGA R: CAGTTCCGGAGGTAATCTGT

B-actin primers were designed as described by Duvigneau et al. [20]

Statistical calculations were performed with STATISTICA ver. 10 (StatSoft, part of Dell Software, USA). For correlation analysis, Spearman r correlation coefficients and P-values were determined, since these values were not distributed normally.

## Results

### Microarray analysis

Microarray analysis revealed differential gene regulation (FC ≥ 1.5, p-value = 0.05) of 12,518 genes in DAN group, 7335 genes in ILI and 4253 genes in BOR group compared to control group (Table 2). Interestingly, in all groups PRRSV nucleocapsid (N) protein encoding gene was found to be highly expressed. To check the level of expression the FC was calculated and was found to be significantly different in all groups, reaching 37.15 in DAN group, 68.02 in ILI and 203.98 in BOR group. These results corresponded to the peak of viremia measured in serum by qRT-PCR [18].

**Table 2:** Overview of differential gene expression in PBMCs of pigs infected with DAN, ILI and BOR PRRSV strains in regard to PBMCs from mock-infected pigs (FC ≥ 1.5, p-value = 0.05)

Number of genes	Groups		
	DAN	ILI	BOR
Differentially expressed	12,518	7335	4253
UP-regulated	6033	3795	2030
DOWN-regulated	6485	3540	2223

**Fig. 1:**
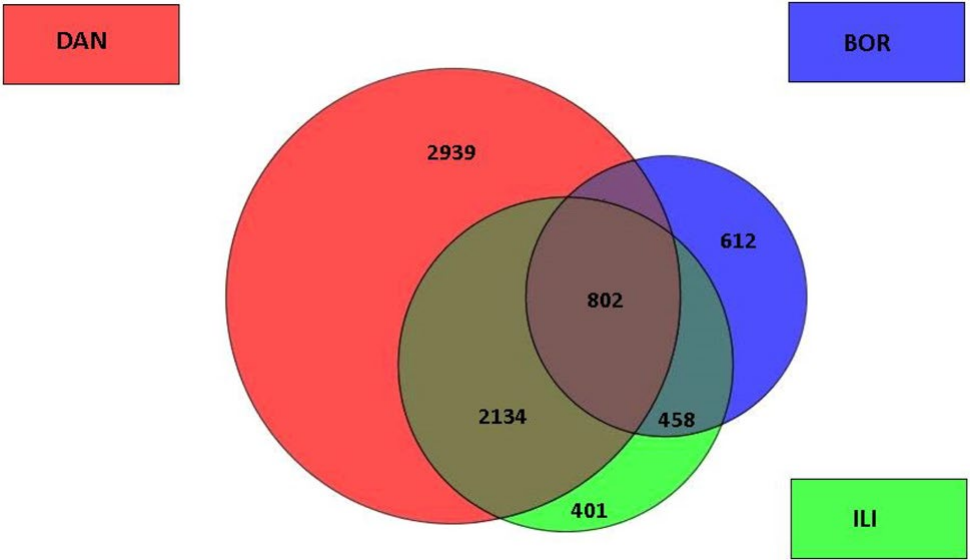
Overview of up-regulated transcripts in DAN (red), BOR (blue) and ILI (green) groups. The numbers in the circles correspond to the number of particular transcripts. Figure was created using the GeneSpring GX10 expression analysis software Agilent Technologies. (Color figure online)

**Fig. 2:**
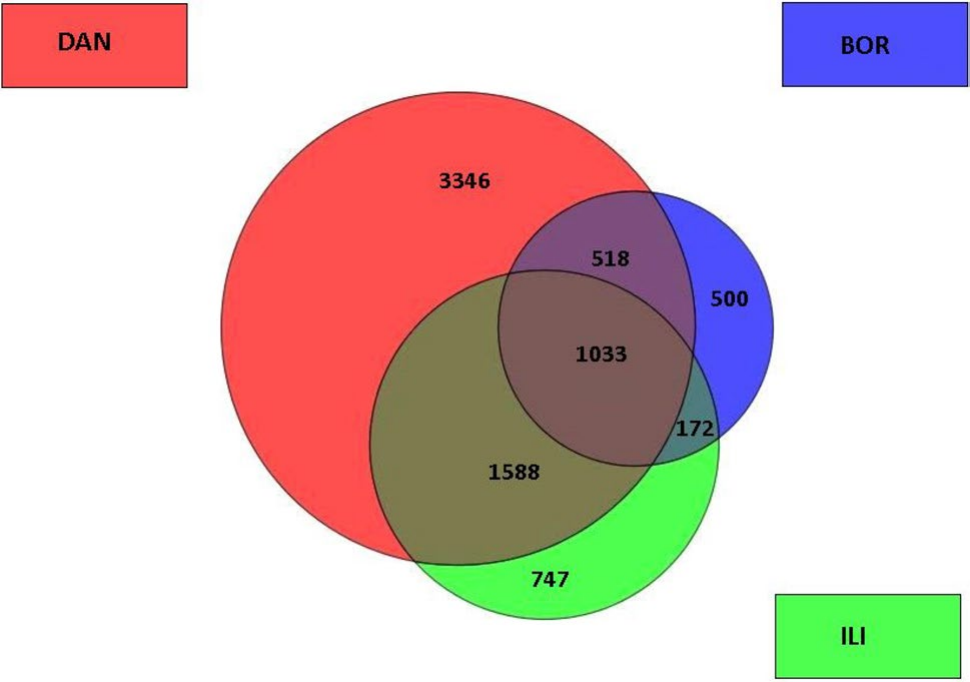
Overview of down-regulated transcripts in DAN (red), BOR (blue) and ILI (green) groups. The numbers in the circles correspond to the number of particular transcripts. Figure was created using the GeneSpring GX10 expression analysis software Agilent Technologies. (Color figure online)

Comparison of the differentially expressed gene lists indicated that 802 genes were up-regulated and 1033 genes were down-regulated in all three tested groups (Figs. 1, 2). Interestingly, the overlap including both down-regulated and up-regulated genes was significantly lower between DAN/BOR and BOR/ILI groups compared to DAN/ILI groups.

The top 50 genes with the highest changes of expression of each tested group PBMCs are indicated in Online Resource 1 (Table S1). The most extensive changes of gene expression (p < 0.5) were observed in cells from BOR infected group of pigs, where four genes showed over 20-fold change in expression (OAS1, GZMA, MX1 and OASL), while in ILI and DAN infected groups only one gene showed more than 20-fold (OAS1). Similarly BOR strain induced over 10-fold change in expression of 11 genes (CXCL-10-, IFIT3, EDN1, RNASE4, IFIT2, PPBP, OAS2, ACTA, CALD1, LHFPL1), compared to five genes in ILI group (GZMA, MX1, OATV1, CXCL-10, IFIT3) and only of four genes in DAN group (GZMA, MBOAT4, TGM5, CXCL-10).

### Functional analysis of PBMCs gene responses to PRRSV strains

The top 5 canonical pathways involving up- and down-regulated genes in each group are presented in Tables 3 and 4. Pathway analysis performed with up-regulated genes demonstrated that one pathway was common for all groups (*Agranulocytes Adhesion and Diapedesis*), and another two canonical pathways were the same in ILI and BOR groups (*Granulocyte Adhesion and Diapedesis, Leukocytes Extravasation Signaling)* (Table 3). Interestingly the top pathway in both ILI and BOR group was the same (*Agranulocytes Adhesion and Diapedesis)*, while in DAN group *Acute Phase Response Signaling* was the top process involving the highest number of up-regulated genes. On the other hand, when down-regulated genes were analysed, two pathways were common in all groups DAN, ILI and BOR (*EIF2 Signaling* and *Regulation of eIF4 and p70S6K Signaling*), one (*Glucocorticoid Receptor Signaling*) was identified in DAN and ILI groups and another one *mTOR signalling* process was noted in DAN and BOR groups (Table 4). Additional analysis was performed to determine top 5 canonical pathways involved in immunological processes (Tables 5, 6). Most of the top canonical pathways involved in immunological processes were the same as in previous analysis. *Agranulocyte Adhesion and Diapedesis* were the pathways involving the highest number of up-regulated genes in ILI and BOR groups, but was also second on the list in DAN group (Table 5). Two pathways observed to be common only for BOR and ILI group were: *Granulocyte Adhesion and Diapedesis* and *Leukocyte Extravasation Signaling. Production of Nitric Oxide and Reactive Oxygen Species in Macrophages* was the process noted as common between DAN and BOR groups, while *Interferon Signaling* was the pathway noted in both, DAN and ILI groups. When analysis considered only down-regulated genes (Table 6), there was only one top immunological pathway found in all three groups (*B Cell Receptor Signaling*). Another two pathways: *NF-κB Signaling* and *CD40 Signaling*, were noted in DAN and ILI groups while *PI3K Signaling in B Lymphocytes* was identified in both BOR and ILI groups. Additionally, one pathway *Production of Nitric Oxide and Reactive Oxygen Species in Macrophages* was noted in DAN group as one of the top canonical pathways in the analyses of up- and down-regulated genes. Interestingly genes with the highest FC values were involved in: *Interferon Signaling Network* (OAS1, MX1, IFIT3, CXCL10, OAS2) *and Toll-like Receptor Signaling Network* (FOS, IL1RN, MYD88, TLR6).

**Table 3:** Top canonical pathways of up-regulated genes as demonstrated by interactive pathway analysis (IPA)

DAN	ILI	BOR
1. Acute phase response signaling 8.43E−11^a^, 36/169 (0.213)^b^	1. Agranulocyte adhesion and diapedesis 2.21E−16, 40/189 (0.212)	1. Agranulocyte adhesion and diapedesis 3.52E−12, 27/189 (0.143)
2. FXR/RXR activation 1.78E−10, 30/126 (0.238)	2. Granulocyte adhesion and diapedesis 3.93E−12, 33/177 (0.186)	2. Integrin signaling 2.81E−09, 25/219 (0.114)
3. Agranulocyte adhesion and diapedesis 9.04E−08, 33/189 (0.175)	3. Leukocyte extravasation signaling 1.81E−09, 32/210 (0.152)	3. Granulocyte adhesion and diapedesis 5.19E−09, 22/177 (0.124)
4. LXR/RXR activation 1.18E−07, 25/121 (0.207)	4. Interferon signaling 2.82E−09, 13/36 (0.361)	4. Leukocyte extravasation signaling 5.71E−09, 24/210 (0.114)
5. Tight junction signaling 1.81E−07, 30/167 (0.18)	5. iNOS signaling 4.52E−08, 13/44 (0.295)	5. ILK signaling 7.14E−09, 23/196 (0.117)

^a^*p*-Value

^b^Pathway ratio

**Table 4:** Top canonical pathways of down-regulated genes as demonstrated by interactive pathway analysis (IPA)

DAN	ILI	BOR
1. Glucocorticoid receptor signaling 1.24E−12^a^, 57/287 (0.199)^b^	1. Integrin signaling 4.39E−06, 23/219 (0.105)	1. EIF2 signaling 1.56E−10, 26/194 (0.134)
2. Regulation of eIF4 and p70S6K signaling 3.70E−12, 39/157 (0.248)	2. Glucocorticoid receptor Signaling 5.45E−06, 27/287 (0.094)	2. Regulation of eIF4 and p70S6K signaling 2.38E−07, 19/157 (0.121)
3. mTOR signaling 1.56E−10, 42/199 (0.211)	3. EIF2 signaling 7.08E−06, 21/194 (0.108)	3. mTOR signaling 2.29E−06, 20/199 (0.101)
4. Production of nitric oxide and reactive oxygen species in macrophages 8.85E−09, 38/193 (0.197)	4. Regulation of eIF4 and p70S6K signaling 1.44E−05, 18/157 (0.115)	4. Cell cycle: G1/S checkpoint regulation 6.08E−04, 8/64 (0.125)
5. EIF2 signaling 1.03E−08, 38/194 (0.196)	5. Adipogenesis pathway 9.61E−05, 15/134 (0.112)	5. Antiproliferative role of TOB in T cell signaling 9.08E−04, 5/26 (0.192)

^a^*p*-Value

^b^Pathway ratio

**Table 5:** Top canonical pathways of up-regulated genes involved in immunological processes as demonstrated by interactive pathway analysis (IPA)

DAN	ILI	BOR
1. Acute phase response signaling 1.94E−05^a^, 19/200 (0.095)^b^	1. Agranulocyte adhesion and diapedesis 8.29E−10, 43/189 (0.228)	1. Agranulocyte adhesion and diapedesis 3.52E−12, 27/189 (0.143)
2. Agranulocyte adhesion and diapedesis 1.95E−05, 15/133 (0.113)	2. Granulocyte adhesion and diapedesis 1.28E−07, 37/177 (0.209)	2. Granulocyte adhesion and diapedesis 5.19E−09, 22/177 (0.124)
3. Interferon signaling 5.66E−05, 17/180 (0.308)	3. Leukocyte extravasation signaling 3.07E−07, 39/198 (0.197)	3. Leukocyte extravasation signaling 5.71E−09, 24/210 (0.114)
4. Clathrin-mediated endocytosis signaling 2.22E−04, 10/81 (0.123)	4. iNOS signaling 1.29E−06, 15/44 (0.341)	4. Production of nitric oxide and reactive oxygen species in macrophages 1.2E−07, 21/193 (0.109)
5. Production of nitric oxide and reactive oxygen species in macrophages 3.19E−05, 28/193 (0.145)	5. Interferon signaling 1.48E−06, 13/34 (0.382)	5. Caveolar-mediated endocytosis signaling 5.82E−07, 12/71 (0.169)

^a^*p*-Value

^b^Pathway ratio

**Table 6:** Top canonical pathways of down-regulated genes involved in immunological processes as demonstrated by interactive pathway analysis (IPA)

DAN	ILI	BOR
1. Production of nitric oxide and reactive oxygen species in macrophages 8.85E−08^a^, 38/193 (0.197)^b^	1. Regulation of IL-2 expression in activated and anergic T lymphocytes 5.16E−04, 10/79 (0.127)	1. Antiproliferative role of TOB in T cell signaling 9.08E−04, 5/26 (0.192)
2. B cell receptor signaling 2.97E−08, 36/185 (0.195)	2. PI3K signaling in B lymphocytes 7.06E−04, 13/128 (0.102)	2. PI3K signaling in B lymphocytes 5.04E−03, 10/128 (0.078)
3. IL-15 signaling 4.69E−08, 21/76 (0.276)	3. NF-κB signaling 8.02E−04, 16/180 (0.089)	3. Production of nitric oxide and reactive oxygen species in macrophages 1.33E−02, 12/193 (0.062)
4. NF-κB signaling 4.7E−08, 35/180 (0.194)	4. B cell receptor signaling 1.07E−03, 16/185 (0.086)	4. T helper cell differentiation 1.91E−02, 6/71 (0.085)
5. CD40 signaling 7.68E−08, 21/78 (0.269)	5. CD40 signaling 1.89E−03, 9/78 (0.115)	5. B cell receptor signaling 2.33E−02, 11/185 (0.059)

^a^*p*-Value

^b^Pathway ratio

### Comparison analysis of canonical signaling pathways activation state induced by BOR, ILI and DAN infection

**Fig. 3:**
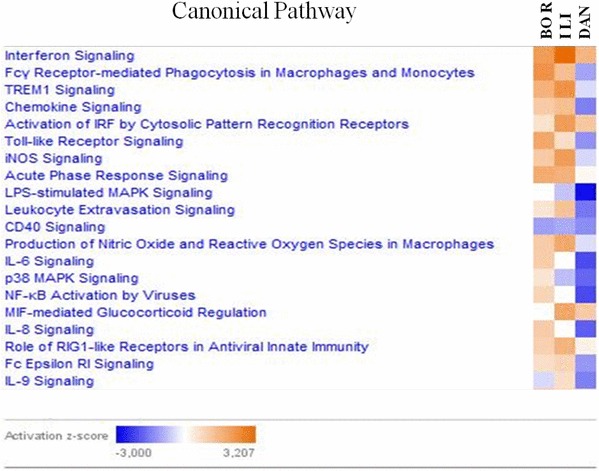
IPA comparison analysis of canonical signaling pathways activation state induced by BOR, ILI and DAN infection. Pathways shaded orange were up-regulated, in blue were down-regulated, in white are those which did not yield any changes in activity. Range of activation z-score is also depicted in the figure. (Color figure online)

IPA was also used to liken the patterns of immune dysregulation observed after infection with three different PRRSV strains. The comparison analysis of both up- and down-regulated genes simultaneously from three groups of infected animals was carried out to gain a global outlook of the canonical pathways that were modulated. The results were presented as a heat map based on activation z-score (Fig. 3), which represents the bias in gene regulation that predicting whether the particular pathway is in an activated or inactivated state. In general, most of the processes were down-regulated in DAN group, in contrast to ILI and BOR. More similarities were observed between ILI and BOR groups, although there were some pathways regulated differently in both groups. From 20 canonical pathways presented in Fig. 3 the pathways with applicable z-score were chosen for further analysis. Three of the top pathways similarly activated in both ILI and BOR groups and inhibited in DAN group were *Fcχ Receptor -Mediated Phagocytosis in Macrophages and Monocytes, TREM1 signaling* and *Chemokine Signaling*. Different regulation predicted by IPA in each of three groups was noted for *IL-6 Signaling*, being in activated state in BOR group, non-altered in ILI group and inhibited in DAN group. The *p38 MAPK Signaling* pathway was activated in BOR group, while in ILI and DAN groups showed different levels of inactivation. While, *IL-8 Signaling*, was associated with positive z-score in BOR group, in ILI z-score was equal to zero and in DAN group it reached a negative value.

### Quantitative RT-qPCR analysis of selected genes

In order to confirm microarray analysis after infection with three different PRRSV strains, six genes involved in immune response were selected to be analysed by RT-qPCR. Spearman’s correlation analysis performed to compare the results of both, microarray and RT-qPCR analyses, revealed strong correlation (R = 0.878225, p-value = 0.000002) between fold changes of selected genes determined by both methods (Table 7). Such concordance between the expression values determined by two methods supports the reliability of the observed expression differences and allows accurate interpretation of obtained data. Furthermore, statistical analysis confirmed very strong correlation (R = 0.831, p-value = 0.001) between individual samples and pooled mRNA quantitation what supported the validity of the pooling strategy applied in this study (Online Resource 2—Table S2) [22].

**Table 7:** Comparison of gene expression changes observed in microarray analysis and RT-qPCR

Gene	PRRSV strain	Fold change	
		Microarray	RT-qPCR
OAS1	DAN	24.12	10.17
	ILI	26.91	50.88
	BOR	35.24	72.11
CXCL2	DAN	− 2.49	− 3.85
	ILI	2.93	9.57
	BOR	5.46	4.28
Il-8	DAN	− 2.75	− 3.70
	ILI	2.24	1.25
	BOR	5.33	1.70
CXCL10	DAN	12.28	24.34
	ILI	10.30	28.06
	BOR	18.80	15.49
FOS	DAN	− 2.90	− 5.32
	ILI	− 2.10	− 2.40
	BOR	− 3.49	− 2.13
IL-4	DAN	− 3.89	− 5.70
	ILI	− 3.22	− 15.60
	BOR	− 2.04	− 18.50

## Discussion

The very high genetic diversity of porcine reproductive and respiratory syndrome virus, as well as its ability to strongly interfere, modulate or inhibit numerous processes during the development of both innate and adaptive immunity, makes it a difficult subject for investigation. Despite many complex studies carried out over the years, understanding of the disease is still far from complete. The answer to such a challenge might be the use of high-throughput analysis tools, like DNA microarrays or deep sequencing, to study transcriptional response of the host after virus infection. Most of such studies included only Type 2 PRRSV strains [23–25], while only single ones enrolled Type 1 strains [26]. Even recently published data on the virulence of subtype 2 strains of Type 1 showed that some of those strains may characterize with high pathogenicity which implies distinct mechanisms shaping the course of infection [18]. However, transcriptional response to infection by subtype 2 strains of Type 1 PRRSV was not studied before.

The aim of a present study was to shed a light on the host-virus interactions during the course of infection with PRRSV strains of commonly present in Central and Western Europe subtype 1 and much less studied subtype 2 strains. The biological material analysed in the present study was collected in the first animal experiment with the use of such strains [18]. Changes in expression of genes involved in immunological processes were evaluated in each infected group of pigs and compared to control pigs.

The demonstration of clear variations in the number of differentially expressed genes in particular groups of animals between BOR infected group and other two groups ILI and DAN indicates that various number of genes were involved in particular processes.

*Interferon Signalling* pathway was significantly associated to up-regulated genes in DAN and ILI group, but not in BOR group. Previous reports showed that PRRSV proteins, including nsp1, nsp2, nsp11, and N, have been identified and characterized as IFN antagonists [27, 28]. Nsp1 has been considered as multifunctional protein regulating type I IFN responses [29–32], and nsp11 and N protein have been described to suppress IFN-β induction by antagonizing IRF3 activation [33, 34]. Interestingly, we did not observe up-regulation of *Interferon Signalling* pathway in BOR group, where the expression of N protein encoding gene reached the highest level.

On the other hand, several genes showing very high increase of expression in all or particular tested groups of animals (Table S1) have been identified as interferon stimulated genes (ISGs): OAS 1 (2′-5′-oligoadenylate synthase 1), MX1 (Myxovirus influenza virus resistance), IFIT1 (interferon-induced protein with tetratricopeptide repeats 1), IFIT2 (interferon-induced protein with tetratricopeptide repeats 2), IFIT3 (interferon-induced protein with tetratricopeptide repeats 3) and ISG15, what shows that all PRRSV strains used in the study activated interferon-induced antiviral response to some degree.

The best studied ISGs so far are OAS1, MX1, IFIT1, ISG15, RNaseL and PKR [35]. Previous reports described already some interactions between PRRSV and ISGs, like inhibition of ISIG15 [36] and PKR [37] functions. Unfortunately, still little is known about particular interactions between PRRSV and ISGs like OAS, IFIT1 or Mx1. Recently Badaoui et al. [38] described up-regulation of OAS1 expression in LV-infected PAMs. In addition, Zhao and co-workers [39] observed in an in vitro system that PRRSV infection led to induction of OAS1, while knockdown of endogenous OAS1 increased the PRRSV mRNA level. Interestingly, our data strongly support this finding in vivo, since the increase of OAS1 expression ranging from FC of 24.121 in DAN group, 26.910 in ILI up to 35.236 in BOR group, was correlated with the strength of particular PRRSV strain replication measured by N protein encoding gene expression. Although the detailed role of OAS1 gene in PRRSV infection is not yet recognized, the results may support a direct restriction function of this gene to virus replication. The function of OAS in the context of PRRSV infection may be similar to this described in other viral infections, where OAS proteins have been identified as enzymes sensing exogenous nucleic acid and initiate antiviral pathways. In our analysis also other genes from OAS family were observed to be strongly up-regulated. OAS2 gene showed increased expression in BOR and ILI groups, while OASL gene was found to be up-regulated in all three groups of infected animals. Those observations suggest the involvement of OAS proteins in various mechanisms engaged in the defense against PRRSV infection, also observed in studies of other viral infections [40–42].

Another ISG which was found to be highly up-regulated, especially in BOR group (FC = 22.739) and ILI (FC = 12.287), but also in DAN group (FC = 5.812) was Mx1 gene, encoding dynamin-like GTPase involved in the innate host defense against RNA viruses [43]. Recently Overend and co-workers showed that Mx1 expression by infected PAMs was generally correlated with IFNβ production [44]. Additionally, recent studies focused on the possibility to use of Mx1 gene as a potential DNA marker for PRRS resistance in pigs [45].

Further genes regarded as ISGs are those encoding interferon-induced proteins with tetratricopeptide repeats: 1, 2 and 3 (IFIT1, IFIT2 and IFIT3). Transcription of IFIT genes is triggered usually in the case of viral and bacterial infections, mostly by Type I IFNs (IFN-α/β) and type III IFNs (IFN-λs) [46, 47]. Independently, IFIT genes are induced in cells infected with RNA viruses which are sensed by pattern recognition receptors [48]. In the presented study increased expression of IFIT1 was noted in DAN (FC = 5.313) and ILI (FC = 3.983) groups, while IFIT2 up-regulation was observed in BOR (FC = 11.521) and ILI (FC = 5.432) groups, as well as IFIT3, which expression increase reached FC = 17.080 in BOR, FC = 10.152 in ILI and FC = 3.245 in DAN group. Interestingly, up to now only IFIT3 was linked to PRRSV infection as an important modulator of innate immunity inhibiting virus replication in MARC-145 cells by induction of IFN-β [49]. In our study, the profile of IFITs expression differed between subtype 1 strain and subtype 2 strains, thus raising the question whether the same mechanisms are utilized during infection with PRRSV strains by those genetic groups. The specific role of IFIT1 and IFIT2 is binding single-stranded RNA, thereby acting as a sensor of viral single-stranded RNAs and inhibiting expression of viral messenger RNA [50, 51]. Some differences in antiviral activity between IFIT proteins were previously described for human parainfluenza virus type 3 [52]. Based on those observations it seems that there are some individual features, like distinct tertiary structures, which allow IFIT proteins to bind different partners and selectively affect host-virus interactions.

At the time of blood collection at 7 dpi, the inflammatory response was already induced. One of the genes which showed a high increase of expression in all three infected groups of animals was CXCL10 (chemokine (C-X-C motif) ligand 10). Its FC ranged from 10.297 in ILI group, to 12.280 in DAN and 18.799 in BOR group. This gene encodes one of proinflammatory chemokine CXCL10 attracting leukocytes to the site of infection [53]. CXCL10 overexpression has been already observed for highly pathogenic PRRSV (HP-PRRSV) isolate [54] and other Type 2 strains [55, 56]. Previous studies showed that expression of proinflammatory cytokines like interleukin 1, 6 or tumor necrosis factor during PRRSV infection corresponds to the severity of infection [57]. In presented study the overexpression of CXCL10 reached the highest level in BOR group and remained at a similar level in both DAN and ILI groups, which corresponds with the most severe clinical outcome of infection observed in BOR-infected pigs [18].

The other gene which was found to be highly up-regulated in all three groups, GZMA (Granzyme A), with FC of 26.066 in BOR, 15.222 in ILI and 19.020 in DAN group, is an abundant protease expressed in all cytotoxic T-cells and NK-cells. GZMA induces caspase-independent cell death with morphological features of apoptosis, when delivered into the target cell through the immunological synapse [58]. In another study the apoptotic cells were found both in B- and T-cell areas of lymphoid organs, suggesting that the apoptosis might play a role in the impairment of the host immune response during PRRSV infection [59]. It is not excluded that in addition to a caspase pathway of apoptosis also caspase-independent mechanism of immunological cells death can be used by PRRSV to debilitate immunological answer to infection, what could explain high up-regulation of GZMA gene expression observed in our analysis.

Comparison of the pathways associated with up- or down-regulated genes between groups of animals infected with particular PRRSV strains revealed some difference indicating that particular strains may utilize variable mechanism to interact with the host.

*Acute Phase Response* was the most significant up-regulated pathway (p-value = 8.43E−11) in a group of piglets infected with subtype 1 strain DAN, while less significant up-regulation was observed in BOR (p-value = 2.24E−05) and ILI (p-value = 5.52E−05) groups for the same pathway at this time post infection. This observation may indicate the differences in the infection progress or some time shift in the course of infection. In both groups infected with subtype 2 strains, BOR and ILI *Agranulocytes Adhesion and Diapedesis* was the most significant up-regulated pathway, followed by *Granulocyte Adhesion and Diapedesis* and *Leukocyte Extravasation Signaling* pathways. This result is not unexpected since an inflammatory response, induced by infection, triggers the movement of leukocytes into body tissue towards the invader. Interestingly, the results of IPA analysis were comparable between both groups, when only immunological processes were considered.

The *Integrin Signaling* pathway has been clearly associated with up-regulated genes in BOR group together with *ILK (integrin-like) signaling* pathway. On the other hand, *Integrin Signaling* pathway was strongly associated with down-regulated genes in ILI group. Integrins are cell surface glycoproteins involved in cell–cell and cell–extracellular matrix interactions, inducing signalling across the cell membrane to regulate cell proliferation, activation, migration and homeostasis [60]. In a recent study, integrins were shown to be involved in sensing of PRRSV infected macrophages by plasmacytoid dendritic cells, which stimulated production of INF-α [61]. Such mechanism seems to allow the host counteract PRRSV strategies aiming the suppression of type I INF induction.

One of the top five pathways identified as up-regulated in DAN group was *Tight Junction Signalling*. Such observation is analogous to the results obtained by Wysocki and co-workers, who also observed the up-regulation of this pathway in lungs of PRRSV-infected pigs [62]. Tight junctions are specialized membrane domains, which maintain adjacent cells close enough to avoid uncontrolled passage of small molecules, microorganisms and cells, across the paracellular space [63, 64]. They are also involved in regulation of some cellular processes, including polarization, proliferation, differentiation and gene expression [65–67]. Our results suggest that also PRRSV may hijack tight junctions to use cellular machinery to support its own replication. It has been already reported that some viruses are able to regulate the expression or localization of tight junctions proteins to induce cell transformation or make their exit process more efficient. Reports evidenced the importance of tight junction for the infection of different viruses, including reoviruses, influenza virus or human immunodeficiency virus 1 [68–71].

Among the most significant pathways associated with down-regulated genes the *Eukaryotic Translation Initiation Factor 2* (*EIF2*) *Signaling and Regulation of Eucaryotic Translation Initiation Factor 4* *(eIF4)* and *p70S6K Signaling* were identified in each tested group. The coincident down-regulation of mTOR Signalling in DAN group and *PI3K Signaling in B Lymphocytes* in ILI and BOR groups indicate the clear influence on inhibition of cellular protein synthesis. This picture, clearly observed in ILI and DAN groups, is known to be one of the hallmarks of interferon signaling [72]. A similar picture was observed by Wilkinson and co-workers in pigs infected with PRRSV Type 2 strain [73]. Additionally, *Cell Cycle: G1/S Checkpoint Regulation* pathway controlling the passage of the cells from the G1 into the DNA synthesis (S) phase, was associated with down-regulated genes in BOR group. Zhou et al. showed already that the genes relevant to cell cycle and DNA replication can be regulated by highly pathogenic PRRSV [74]. Similarly, Sun and co-workers noticed in microarray experiment that PRRSV nsp11 is able to regulate cell cycle and DNA replication and by in vitro experiment proved that nsp11 induced the delay of cell cycle progression at the S phase. Such cell cycle arrest may be beneficial for the virus, since it can redirect cellular replicative machinery for viral replication, as can be observed for other DNA and RNA viruses [75].

Among pathways affected significantly by down-regulated genes in DAN and ILI group also *CD40 Signaling* was identified, and in all groups *B cell receptor Signaling* was down-regulated. Ligation of CD40 on the surface of dendritic cells controls the production of particular proinflammatory cytokines (IL-8, MIP-1α, TNF-α and IL-12), while its ligation on monocytes is required for stimulation of production of IL-1α, IL-1β, TNF-α, IL-6, and IL-8, and in the rescue of circulating monocytes from apoptosis. The impairment of this pathway affects simultaneously both cellular and humoral immune response. The impairment of B cell receptor signaling strongly affects humoral immune response, since signals propagated through the B cell antigen receptor (BCR) are crucial to the development, survival and activation of B lymphocytes. Furthermore, it is also linked with stimulation of nuclear factor kappa B (NFκB) and PI3K/AKT signaling pathways resulting in the nuclear accumulation of transcription factors and enhancement of protein synthesis and therefore its down-regulation impairs mentioned processes, accompanying already mentioned pathways, EIF2 and EIF4 [76]. In BOR group, additionally, *Antiproliferative Role of TOB in T Cell Signaling* and *T Helper Cell Differentiation* processes were found to be significantly associated with down-regulated genes. A transducer of ERBB2 (TOB) is a negative regulator of T cell proliferation and cytokine transcription, which is constitutively expressed in unstimulated peripheral blood T lymphocytes and selectively expressed in anergic T cells. Down-regulation of TOB is necessary for T cell activation what is crucial during infection [77]. But when this whole process undergoes down-regulation, together with *T Helper Cell Differentiation* process, it changes the situation and heavily impairs cellular immune response and cytokines signaling, crucial in the face of infection [78].

Comparison analysis of canonical signaling pathways activation state induced by BOR, ILI and DAN infection showed some differences between strains. *Fcγ Receptor-mediated Phagocytosis in Macrophages and Monocytes, TREM 1 signaling* and *Chemokine Signaling* pathways were activated in both strains from subtype 2 (BOR and ILI), while in subtype 1 strain DAN their inhibition was observed. *Fcγ Receptor-mediated Phagocytosis in Macrophages and Monocytes* plays a role in defense against invading bacteria. Infection with PRRSV may sensitize pigs to secondary bacterial infection. It was shown that expression of FcγRIIB was up-regulated post-infection with PRRSV strains HN07-1 and BJ-4 but an expression of FcγRIIIA receptor was inhibited, what in consequence could suppress the phagocytosis of granulocytes [79]. On the other hand, FcγR-mediated activation of monocyte derived macrophages (MDM) is a potent mechanism of HIV-1 suppression [80]. The activation of *Fcγ Receptor-mediated Phagocytosis in Macrophages and Monocytes* pathway after BOR and ILI infection may reflect the host attempt to control viral replication. Although the whole pathway is in an inactivated state in the DAN-infected group, we observed up-regulation of Fc gamma receptors (subtypes FcγR1A, FC = 3.7).

*TREM 1 signaling* leads to the induction of inflammatory processes such as cytokine production, degranulation of neutrophils and phagocytosis. Badaoui et al. [38] reported the activation of *TREM1 signaling* pathway in response to the infection with highly virulent East European PRRSV strain Lena (subtype 3 of Type 1). Similarly, an increase of activity in TREM 1 pathway was noted for ILI and BOR strains. Furthermore, in infection with Lena as well as ILI and BOR strains, the expression of IL-8 and TLR4 was up-regulated. This finding could suggest common mechanism playing role in the infection with genetically different East European strains and their higher pathogenicity compared to classical PRRSV strains present in Central and Western European countries.

Further IPA analysis revealed an additive effect of immunity related genes engaged in *Chemokine Signaling* pathway. An activation of this pathway was observed previously in the transcriptomic analysis of PBMCs after PRRSV vaccination [81]. Particularly interesting results were described for CCL4 cytokine (MIP-1β), recently investigated in several PRRSV studies. Miller et al. [82] and López-Fuertes et al. [83] observed that the level of transcripts encoding CCL4 declined in porcine alveolar macrophages following infection with both Type 2 strain VR-2332 and European Type 1 PRRSV isolate 5710. In our study we observed the opposite effect and up-regulated expression of CCL-4 in BOR (FC = 2.5) and ILI (FC = 1.8) groups. The same additive effect for this inflammatory mediator was noted in bone marrow-derived dendritic cells BMDCs pre-infected with PRRSV (IAF-Klop PRRSV genotype 2 strain) after a subsequent infection with *S. suis* (wild-type virulent *S. suis* serotype 2 strain P1/7) [84]. CCL-4 is considered as the most potent chemoattractant and mediator of virus-induced inflammation in vivo [85], thus acitivation of *Chemokine Signaling* pathway and up-regulation of CCL-4 can contribute to the higher virulence of BOR and ILI strains compared to DAN observed in an animal experiment.

Three pathways *IL-6 Signaling, IL-8 Signaling and p38 MAPK Signaling* were characterized as up-regulated only in BOR group and non-altered or down-regulated in two remaining strains. Interleukin 6 is a regulator of acute-phase response and also a lymphocyte stimulator factor, interleukin 8 plays a central role in inflammation process, while p38 MAPK pathway is known to be necessary for induction of different inflammatory cytokines in respiratory viral infections [86] and have been confirmed to be activated by PRRSV [87, 88]. The activation state of signaling pathways of two pro-inflammatory cytokines, *IL*-*6* and *IL*-*8*, as well as induction of the *p38 MAPK* pathway, suggests potential mechanisms responsible for the highest virulence of BOR strain observed in the experimental infection study [18].

Although the samples for microarray analysis were pooled, strong correlation of RT-qPCR results between pooled and individual samples confirmed the validity of obtained results. Moreover, the results of the present study were consistent with the results of an experimental infection study, where the group infected with BOR strain developed acute clinical symptoms, in contrast to medium and mild severity in case of ILI and DAN strains, respectively [18]. The highest FC values obtained in BOR group for multiple genes (OAS1, Mx1, IFIT2, IFIT3, CXCL10, GZMA) involved in a range of immunological processes indicate the most pronounced inflammatory response. Also, BOR strain seems to have a higher general influence on the cells’ metabolic processes and signaling pathways (down-regulation of cell cycle related *G1/S Checkpoint* Pathway, up-regulation of *Integrin* and *Integrin-like Signaling* Pathways, dysregulation of TOB activity, lack of up-regulation of *Interferon Signaling* Pathway in contrast to ILI and DAN strains).

Presented results, referring to different transcriptional profiles of pigs infected with three PRRSV strains, create an important platform for further studies on pathogenicity and immune mechanisms used by PRRSV strains of subtype 2 and 1 to sabotage host immune activation. However, more studies are necessary to identify the full spectrum of pathways influenced by particular strains of PRRSV, especially in the context of extensive genetic variability observed within PRRSV.

## Electronic supplementary material

Below is the link to the electronic supplementary material.

Supplementary material 1 (XLS 27 KB)

Supplementary material 2 (XLS 23 KB)

## References

[1] Murtaugh MP, Stadejek T, Abrahante J (2010). The ever-expanding diversity of porcine reproductive and respiratory syndrome virus. Virus Res.

[2] Stadejek T, Stankevicius A, Murtaugh MP (2013). Molecular evolution of PRRSV in Europe: current state of play. Vet Microbiol.

[3] Shi M, Lam TT, Hon CC (2010). Phylogeny-based evolutionary, demographical, and geographical dissection of North American type 2 porcine reproductive and respiratory syndrome viruses. J Virol.

[4] Drigo M, Franzo G, Gigli A (2014). The impact of porcine reproductive and respiratory syndrome virus genetic heterogeneity on molecular assay performances. J Virol Methods.

[5] Martín-Valls GE, Kvisgaard LK, Tello M (2014). Analysis of ORF5 and full-length genome sequences of porcine reproductive and respiratory syndrome virus isolates of genotypes 1 and 2 retrieved worldwide provides evidence that recombination is a common phenomenon and may produce mosaic isolates. J Virol.

[6] Lu WH, Tun HM, Sun BL (2015). Re-emerging of porcine respiratory and reproductive syndrome virus (lineage 3) and increased pathogenicity after genomic recombination with vaccine variant. Vet Microbiol.

[7] Zhao K, Ye C, Chang XB (2015). Importation and recombination are responsible for the latest emergence of highly pathogenic porcine reproductive and respiratory syndrome virus in China. J Virol.

[8] Tian K, Yu X, Zhao T (2007). Emergence of fatal PRRSV variants: unparalleled outbreaks of atypical PRRS in China and molecular dissection of the unique hallmark. PLoS ONE.

[9] Zhou YJ, Hao XF, Tian ZJ (2008). Highly virulent porcine reproductive and respiratory syndrome virus emerged in China. Transbound Emerg Dis.

[10] Karniychuk UU, Geldhof M, Vanhee M (2010). Pathogenesis and antigenic characterization of a new East European subtype 3 porcine reproductive and respiratory syndrome virus isolate. BMC Vet Res.

[11] Morgan SB, Graham SP, Salguero FJ (2013). Increased pathogenicity of European porcine reproductive and respiratory syndrome virus is associated with enhanced adaptive responses and viral clearance. Vet Microbiol.

[12] Weesendorp E, Rebel JM, Popma-De Graaf DJ (2014). Lung pathogenicity of European genotype 3 strain porcine reproductive and respiratory syndrome virus (PRRSV) differs from that of subtype 1 strains. Vet Microbiol.

[13] Sun Y, Han M, Kim C (2012). Interplay between interferon-mediated innate immunity and porcine reproductive and respiratory syndrome virus. Viruses.

[14] Molina RM, Cha SH, Chittick W (2008). Immune response against porcine reproductive and respiratory syndrome virus during acute and chronic infection. Vet Immunol Immunopathol.

[15] Lyoo YS (2015). Porcine reproductive and respiratory syndrome virus vaccine does not fit in classical vaccinology. Clin Exp Vaccine Res.

[16] Weesendorp E, Morgan S, Stockhofe-Zurwieden N (2013). Comparative analysis of immune responses following experimental infection of pigs with European porcine reproductive and respiratory syndrome virus strains of differing virulence. Vet Microbiol.

[17] Weesendorp E, Stockhofe-Zurwieden N, Nauwynck HJ (2016). Characterization of immune responses following homologous reinfection of pigs with European subtype 1 and 3 porcine reproductive and respiratory syndrome virus strains that differ in virulence. Vet Microbiol.

[18] Stadejek T, Larsen LE, Podgórska K (2017). Pathogenicity of three genetically diverse strains of PRRSV Type 1 in specific pathogen free pigs. Vet Microbiol.

[19] Bøtner A, Nielsen J, Bille-Hansen V (1994). Isolation of porcine reproductive and respiratory syndrome (PRRS) virus in a Danish swine herd and experimental infection of pregnant gilts with the virus. Vet Microbiol.

[20] Duvigneau JC, Hartl RT, Groiss S (2005). Quantitative simultaneous multiplex real-time PCR for the detection of porcine cytokines. J Immunol Methods.

[21] Pfaffl MW (2001). A new mathematical model for relative quantification in real-time RT-PCR. Nucleic Acids Res.

[22] Mas VR, Maluf DG, Stravitz R (2004). Hepatocellular carcinoma in HVC-infected patients awaiting liver transplantation: genes involved in tumor progression. Liver Transpl.

[23] Schroyen M, Steibel JP, Koltes JE (2015). Whole blood microarray analysis of pigs showing extreme phenotypes after a porcine reproductive and respiratory syndrome virus infection. BMC Genom.

[24] Nedumpun T, Wongyanin P, Sirisereewan C (2017). Interleukin-1 receptor antagonist (IL-1Ra): an early immunomodulatory cytokine induced by porcine reproductive and respiratory syndrome virus (PRRSV). J Gen Virol.

[25] Wilkinson JM, Bao H, Lading A (2016). Genome-wide analysis of the transcriptional response to porcine reproductive and respiratory syndrome virus infection at the maternal/fetal interface and in the fetus. BMC Genom.

[26] Genini S, Delputte PL, Malinverni R (2008). Genome-wide transcriptional response of primary alveolar macrophages following infection with porcine reproductive and respiratory syndrome virus. J Gen Virol.

[27] Yoo D, Song C, Sun Y (2010). Modulation of host cell responses and evasion strategies for porcine reproductive and respiratory syndrome virus. Virus Res.

[28] Sun Y, Han M, Kim C (2012). Interplay between interferon-mediated innate immunity and porcine reproductive and respiratory syndrome virus. Viruses.

[29] Beura LK, Sarkar SN, Kwon B (2010). Porcine reproductive and respiratory syndrome virus nonstructural protein 1beta modulates host innate immune response by antagonizing IRF3 activation. J Virol.

[30] Chen Z, Lawson S, Sun Z (2010). Identification of two auto-cleavage products of nonstructural protein 1 (nsp1) in porcine reproductive and respiratory syndrome virus infected cells: nsp1 function as interferon antagonist. Virology.

[31] Kim O, Sun Y, Lai FW (2010). Modulation of type I interferon induction by porcine reproductive and respiratory syndrome virus and degradation of CREB-binding protein by non-structural protein 1 in MARC-145 and HeLa cells. Virology.

[32] Beura LK, Subramaniam S, Vu HL (2012). Identification of amino acid residues important for anti-IFN activity of porcine reproductive and respiratory syndrome virus non-structural protein 1. Virology.

[33] Shi X, Wang L, Li X (2011). Endoribonuclease activities of porcine reproductive and respiratory syndrome virus nsp11 was essential for nsp11 to inhibit IFN-beta induction. Mol Immunol.

[34] Sagong M, Lee C (2011). Porcine reproductive and respiratory syndrome virus nucleocapsid protein modulates interferon-β production by inhibiting IRF3 activation in immortalized porcine alveolar macrophages. Arch Virol.

[35] Wang R, Zhang YJ (2014). Antagonizing interferon-mediated immune response by porcine reproductive and respiratory syndrome virus. Biomed Res Int.

[36] Sun Z, Ransburgh R, Snijder EJ (2012). Nonstructural protein 2 of porcine reproductive and respiratory syndrome virus inhibits the antiviral function of interferon-stimulated gene 15. J Virol.

[37] Rowland RRR, Robinson B, Stefanick J (2001). Inhibition of porcine reproductive and respiratory syndrome virus by interferon-gamma and recovery of virus replication with 2-aminopurine. Arch Virol.

[38] Badaoui B, Rutigliano T, Anselmo A (2014). RNA-sequence analysis of primary alveolar macrophages after in vitro infection with porcine reproductive and respiratory syndrome virus strains of differing virulence. PLoS ONE.

[39] Zhao J, Feng N, Li Z (2016). 2′,5′-Oligoadenylate synthetase 1(OAS1) inhibits PRRSV replication in Marc-145 cells. Antivir Res.

[40] Schröder HC, Suhadolnik RJ, Pfleiderer W (1992). (2′-5′) Oligoadenylate and intracellular immunity against retrovirus infection. Int J Biochem.

[41] Kristiansen H, Scherer CA, McVean M (2010). Extracellular 2′-5′ oligoadenylate synthetase stimulates RNase L-independent antiviral activity: a novel mechanism of virus-induced innate immunity. J Virol.

[42] Choi UY, Kang JS, Hwang YS (2015). Oligoadenylate synthase-like (OASL) proteins: dual functions and associations with diseases. Exp Mol Med.

[43] Palm M, Garigliany M-M, Cornet F (2010). Interferon-induced Sus scrofa Mx1 blocks endocytic traffic of incoming influenza A virus particles. Vet Res.

[44] Overend CC, Cui J, Grubman MJ (2017). The activation of the IFNβ induction/signaling pathway in porcine alveolar macrophages by porcine reproductive and respiratory syndrome virus is variable. Vet Res Commun.

[45] Wang SJ, Liu WJ, Sargent CA (2012). Effects of the polymorphisms of Mx1, BAT2 and CXCL12 genes on immunological traits in pigs. Mol Biol Rep.

[46] Der SD, Zhou A, Williams BR (1998). Identification of genes differentially regulated by interferon alpha, beta, or gamma using oligonucleotide arrays. Proc Natl Acad Sci USA.

[47] Kohli A, Zhang X, Yang J (2012). Distinct and overlapping genomic profiles and antiviral effects of Interferon-λ and -α on HCV-infected and noninfected hepatoma cells. J Viral Hepat.

[48] Broz P, Monack DM (2013). Newly described pattern recognition receptors team up against intracellular pathogens. Nat Rev Immunol.

[49] Zhang L, Liu J, Bai J (2013). Poly(I:C) inhibits porcine reproductive and respiratory syndrome virus replication in MARC-145 cells via activation of IFIT3. Antiviral Res.

[50] Terenzi F, Saikia P, Sen GC (2008). Interferon-inducible protein, P56, inhibits HPV DNA replication by binding to the viral protein E1. EMBO J.

[51] Raychoudhuri A, Shrivastava S, Steele R (2011). ISG56 and IFITM1 proteins inhibit hepatitis C virus replication. J Virol.

[52] Rabbani MA, Ribaudo M, Guo JT (2016). Identification of IFN-stimulated gene (ISG) proteins that inhibit human parainfluenza virus type 3. J Virol.

[53] Lusso P (2000). Chemokines and viruses: the dearest enemies. Virology.

[54] Chen XX, Quan R, Guo XK (2014). Up-regulation of pro-inflammatory factors by HP-PRRSV infection in microglia: implications for HP-PRRSV neuropathogenesis. Vet Microbiol.

[55] Yu Y, Wang R, Nan Y (2013). Induction of STAT1 phosphorylation at serine 727 and expression of proinflammatory cytokines by porcine reproductive and respiratory syndrome virus. PLoS ONE.

[56] Xiao S, Jia J, Mo D (2010). Understanding PRRSV infection in porcine lung based on genome-wide transcriptome response identified by deep sequencing. PLoS ONE.

[57] Gomez-Laguna J, Salguero FJ, Barranco I (2010). Cytokine expression by macrophages in the lung of pigs infected with the porcine reproductive and respiratory syndrome virus. J Comp Pathol.

[58] Beresford PJ, Zhang D, Oh DY (2001). Granzyme A activates an endoplasmic reticulum-associated caspase-independent nuclease to induce single-stranded DNA nicks. J Biol Chem.

[59] Gómez-Laguna J, Salguero FJ, Fernández de Marco M (2013). Type porcine reproductive and respiratory syndrome virus infection mediated apoptosis in B- and T-cell areas in lymphoid organs of experimentally infected pigs. Transbound Emerg Dis.

[60] Hynes RO (2002). Integrins: bidirectional, allosteric signaling machines. Cell.

[61] García-Nicolás O, Auray G, Sautter CA (2016). Sensing of porcine reproductive and respiratory syndrome virus-infected macrophages by plasmacytoid dendritic cells. Front Microbiol.

[62] Wysocki M, Chen H, Steibel JP (2012). Identifying putative candidate genes and pathways involved in immune responses to porcine reproductive and respiratory syndrome virus (PRRSV) infection. Anim Genet.

[63] Steed E, Balda MS, Matter K (2010). Dynamics and functions of tight junctions. Trends Cell Biol.

[64] Shen L (2011). Tight junctions on the move: Molecular mechanisms for epithelial barrier regulation. Ann N Y Acad Sci.

[65] Zihni C, Bald MS, Matter K (2014). Signalling at tight junctions during epithelial differentiation and microbial pathogenesis. J Cell Sci.

[66] Gonzalez-Mariscal L, Tapia R, Chamorro D (2008). Crosstalk of tight junction components with signaling pathways. Biochim Biophys Acta.

[67] Tsukita S, Yamazaki Y, Katsuno T (2008). Tight junction-based epithelial microenvironment and cell proliferation. Oncogene.

[68] Boheme KW, Lai CM, Dermody TS (2013). Mechanisms of reovirus bloodstream dissemination. Adv Virus Res.

[69] Golebiewski L, Liu H, Javier RT (2011). The avian influenza virus NS1 ESEV PDZ binding motif associates with Dlg1 and scribble to disrupt cellular tight junctions. J Virol.

[70] Sufiawati I, Tugizov SM (2014). HIV-associated disruption of tight and adherens junctions of oral epithelial cells facilitates HSV-1 infection and spread. PLoS ONE.

[71] Torres-Flores JM, Arias CF (2015). Tight junctions go virus!. Viruses.

[72] Riley FL, Levy HB (1977). Effect of interferon on cellular RNA synthesis and structure. Tex Rep Biol Med.

[73] Wilkinson JM, Ladinig A, Bao H (2016). Differences in whole blood gene expression associated with infection time-course and extent of fetal mortality in a reproductive model of type 2 porcine reproductive and respiratory syndrome virus (PRRSV) infection. PLoS ONE.

[74] Zhou P, Zhai S, Zhou X (2011). Molecular characterization of transcriptome-wide interactions between highly pathogenic porcine reproductive and respiratory syndrome virus and porcine alveolar macrophages in vivo. Int J Biol Sci.

[75] Sun Y, Li D, Giri S (2014). Differential host cell gene expression and regulation of cell cycle progression by nonstructural protein 11 of porcine reproductive and respiratory syndrome virus. Biomed Res Int.

[76] B Cell Receptor Signaling Pathway - Target Explorer. https://targetexplorer.ingenuity.com/pathway/ING/ING:cil. Accessed 27 Nov 2016

[77] Antiproliferative Role of TOB in T Cell Signaling Pathway – Target. https://targetexplorer.ingenuity.com/pathway/ING/ING:5c93d. Accessed 27 Nov 2016

[78] T Helper Cell Differentiation Pathway - Target Explorer. https://targetexplorer.ingenuity.com/pathway/ING/ING:3qbdh. Accessed 27 Nov 2016

[79] Wan B, Qiao S, Li P (2013). Impairment of the antibody-dependent phagocytic function of PMNs through regulation of the FcγRs expression after porcine reproductive and respiratory syndrome virus infection. PLoS ONE.

[80] Perez-Bercoff D, David A, Sudry H (2003). Fcγ receptor-mediated suppression of human immunodeficiency virus type 1 replication in primary human macrophages. J Virol.

[81] Islam MA, Große-Brinkhaus C, Pröll MJ (2016). Deciphering transcriptome profiles of peripheral blood mononuclear cells in response to PRRSV vaccination in pigs. BMC Genom.

[82] Miller LC, Neill JD, Harhay GP (2010). In-depth global analysis of transcript abundance levels in porcine alveolar macrophages following infection with porcine reproductive and respiratory syndrome virus. Adv Virol.

[83] López Fuertes L, Doménech N, Alvarez B (1999). Analysis of cellular immune response in pigs recovered from porcine respiratory and reproductive syndrome infection. Virus Res.

[84] Auray G, Lachance C, Wang Z (2016). Transcriptional analysis of PRRSV-infected porcine dendritic cell response to streptococcus suis infection reveals up-regulation of inflammatory-related genes expression. PLoS ONE.

[85] Salazar-Mather TP, Hokeness KL (2006). Cytokine and chemokine networks: pathways to antiviral defense. Curr Top Microbiol Immunol.

[86] Meusel TR, Imani F (2003). Viral induction of inflammatory cytokines in human epithelial cells follows a p38 mitogen-activated protein kinase-dependent but NF-κB-independent pathway. J Immunol.

[87] Hou J, Wang L, Quan R (2012). Induction of interleukin-10 is dependent on p38 mitogen-activated protein kinase pathway in macrophages infected with porcine reproductive and respiratory syndrome virus. Virol J.

[88] Lee YJ, Lee C (2012). Stress-activated protein kinases are involved in porcine reproductive and respiratory syndrome virus infection and modulate virus-induced cytokine production. Virology.

